# A case report on clinical features, diagnosis, and treatment of rhino‐orbito‐cerebral mucormycosis

**DOI:** 10.1002/iid3.1080

**Published:** 2023-11-09

**Authors:** Jie‐Qiong Ding, Ying Xie

**Affiliations:** ^1^ Department of Opthalmology Shanxi Provincial People's Hospital Taiyuan China

**Keywords:** diabetes mellitus, fungal infection, mucormycosis, rhino‐orbito‐cerebral

## Abstract

**Background:**

Rhino‐orbito‐cerebral mucormycosis (ROCM) is an opportunistic pathogenic fungal disease caused by the fungus mucor, and it is a life‐threatening fungal infection. The ulceration on the skin of the head and neck, accompanied by rhinitis, headache, orbital inflammation, and eyelid edema, should raise a high suspicion of *Mucor* infection in diabetic patients with inadequately controlled blood glucose.

**Case Description:**

The clinical data of a patient with ROCM were analyzed retrospectively, and the clinical features were analyzed. The patient was admitted to the hospital with “diabetic hyperosmotic coma” after presenting with fatigue, poor appetite, and disturbances in consciousness as initial symptoms. After improving relevant examinations, controlling underlying diseases, and administering antifungal treatment, the final clinical outcome was death.

**Conclusion:**

ROCM is more prevalent in patients with uncontrolled diabetes and varied clinical manifestations. The characteristic feature is an eschar‐like necrosis of the local skin or mucosa. The gold criteria for diagnosis are pathology and fungal culture; imaging examination does not reveal any specific manifestations. Early diagnosis and effective treatment are the keys.

## Graphical abstract

Rhino‐orbito‐cerebral mucormycosis is more prevalent in patients with uncontrolled diabetes and varied clinical manifestations. The characteristic feature is an eschar‐like necrosis of the local skin or mucosa. The gold criteria for diagnosis are pathology and fungal culture; imaging examination does not reveal any specific manifestations. Early diagnosis and effective treatment are the keys.

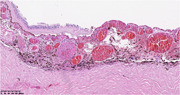

## 1 INTRODUCTION

As the most common fungal infection of Mucoraceae, rhino‐orbito‐cerebral mucormycosis (ROCM) occurs more frequently in patients with diabetic ketosis or acidosis.^1^ ROCM can manifest with symptoms such as nasal congestion, a congested nose, and a headache. Patients may develop high fever, periorbital pain, exophthalmos, limited eye movement, decreased vision, cranial nerve involvement, and even disturbance of consciousness if the infection invades orbital and intracranial regions.^2^ In 1885, Paltauf reported the first case of human mucormycosis,^3^ with an annual incidence rate of up to 1.2 per million people.^4^ *Mucor* is widespread in the natural environment and can infect susceptible hosts through inhalation of *Mucor* spores, ingestion of contaminated food, or skin injury.^5^ In ophthalmology, fungal infections of the nose, orbit and brain are relatively rare. In this case, the eschar‐like necrotic lesion discovered by ophthalmologists during surgery plays a crucial role in the final diagnosis, opening up new ideas for clinical multidisciplinary collaborative diagnosis and treatment.

## 2 CASE DATA

### 2.1 General data

On October 23, 2022, a 51‐year‐old male patient experienced fatigue, poor appetite, drowsiness, restlessness, and thirst without an obvious cause. He subsequently visited multiple hospitals, and on October 28, 2022, he underwent a head MRI at a hospital, which revealed no hemorrhage or infarction. His symptoms worsened without treatment, and he had bouts of unconsciousness and did not respond to vocal stimuli. The following laboratory tests were performed at the hospital: blood glucose 55 mmol/L, blood amylase 238.2 U/L, and lipase 763.7 U/L. The patient received a fluid supplement and symptomatic treatment for “diabetic hyperosmolar coma and acute pancreatitis,” but his symptoms did not improve. He was then transported by ambulance to Shanxi Provincial People's Hospital November 1, 2022. The patient was admitted with a mild coma, an APACHE II score of 13, an inability to respond to calls, an inability to communicate, eyelid proptosis, a severe left eye, a low fever without limb convulsions, and other discomfort. Past medical history: hypertension for more than a decade; denied history of diabetes.

Physical examination: body temperature 38.2°C, blood pressure 120/68 mmHg, acute appearance, moderate nutrition, drowsiness, spontaneous breathing, response to calls, eyes proptosis, severe left eye, left upper ptosis, congestion and edema of the left bulbar conjunctiva, fixed eyeball unable to rotate, dilated pupils measuring 4.5 mm, disappeared direct and indirect light reactions; the right eye is able to open, there is no edema in the bulbar conjunctiva, the pupil is 2.5 mm, and the direct light reaction is sensitive, the indirect light reaction has vanished.

Imaging examination: abdominal color doppler ultrasound: fatty liver; full pancreas; multiple cysts in the left kidney; multiple stones in the left kidney; left hydronephrosis; no obvious abnormality was found in the gallbladder, spleen, right kidney, or portal vein. CT of the head, chest, abdomen, and pelvis: acute pancreatitis, exudative changes around the pancreas, rough intestinal wall adjacent to the descending duodenum; left ureteral pelvic calculi with mild dilatation and hydronephrosis of the proximal ureter and renal pelvis and renal calices; bilateral renal calices stones; right renal cyst; fatty liver; a small amount of ascites; Mild hypostatic inflammation in the bilateral posterior of both lungs; bulla in upper lobe of both lungs; solid nodules in the upper lobe of the left lung; bilateral exophthalmos: subcutaneous density increase and scalp swelling on the top of the left side.

Laboratory tests: blood glucose 20.0 mmol/L, blood routine: white blood cell count 9.26 × 10^9^//L, neutrophil percentage 80.1%; red blood cell count 4.67 × 10; hemoglobin 142 g/L; hematocrit 0.418, platelet count 159 × 10^9^/L; C‐reactive protein 315.00 mg/L; blood chemistry: Alanine aminotransferase 16.73 IU/L, aspartate aminotransferase 23.00 IU/L, albumin 27.73 g/L, total bilirubin 13.32 µmol/L, direct bilirubin 3.07 µmol/L, indirect bilirubin 10.25 µmol/L, inorganic phosphate 0.45 mmol/L, amylase 185.29 IU/L, urea 16.12 mmol/L, serum creatinine 117.2 µmol/L, carbon dioxide (bicarbonate) 15.49 mmol/L, potassium 3.57 mmol/L, sodium 145.45 mmol/L, chlorine 111.82 mmol/L, blood ammonia 26.64 µmol/L, lipase 579.74 IU/L; coagulation function: prothrombin time 12.4 s, activated partial thromboplastin time 32.0 s, fibrinogen 9.228/L: myocardial enzyme: cardiac troponin I < 0.01 ng/mL, creatine kinase isoenzyme 3.87 ng/mL, myoglobin 67.0 ng/ml, N‐terminal pro‐brain natriuretic peptide 370 pg/mL, d‐dimer 5.15 mg/L: blood gas analysis: pH 7.302, partial pressure of carbon dioxide 29.1 mmHg, partial pressure of oxygen 128.8 mmHg, actual carbonate 14.1 mmol/L, standard carbonate 16.2 mmol/L, base excess in extravascular fluid −10.7 mmol/L, base excess in blood −12.3 mmol/L, lactic acid 2.38 mmol/L.

### 2.2 Treatment

**Figure 1:**
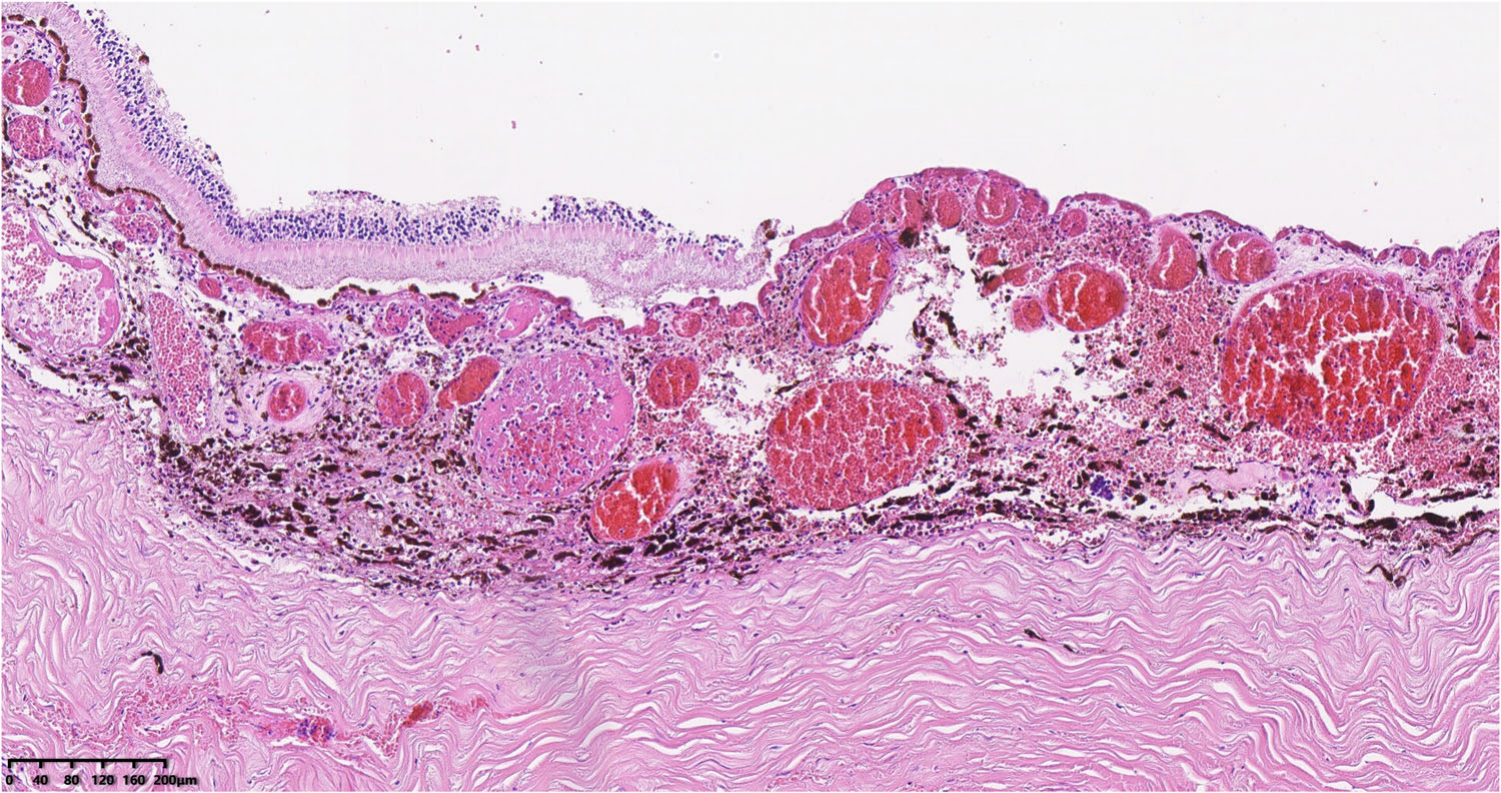
Retinal detachment, massive neutrophil infiltration in choroid layers with vasodilation and congestion, focal necrosis (HE stain X10).

**Figure 2:**
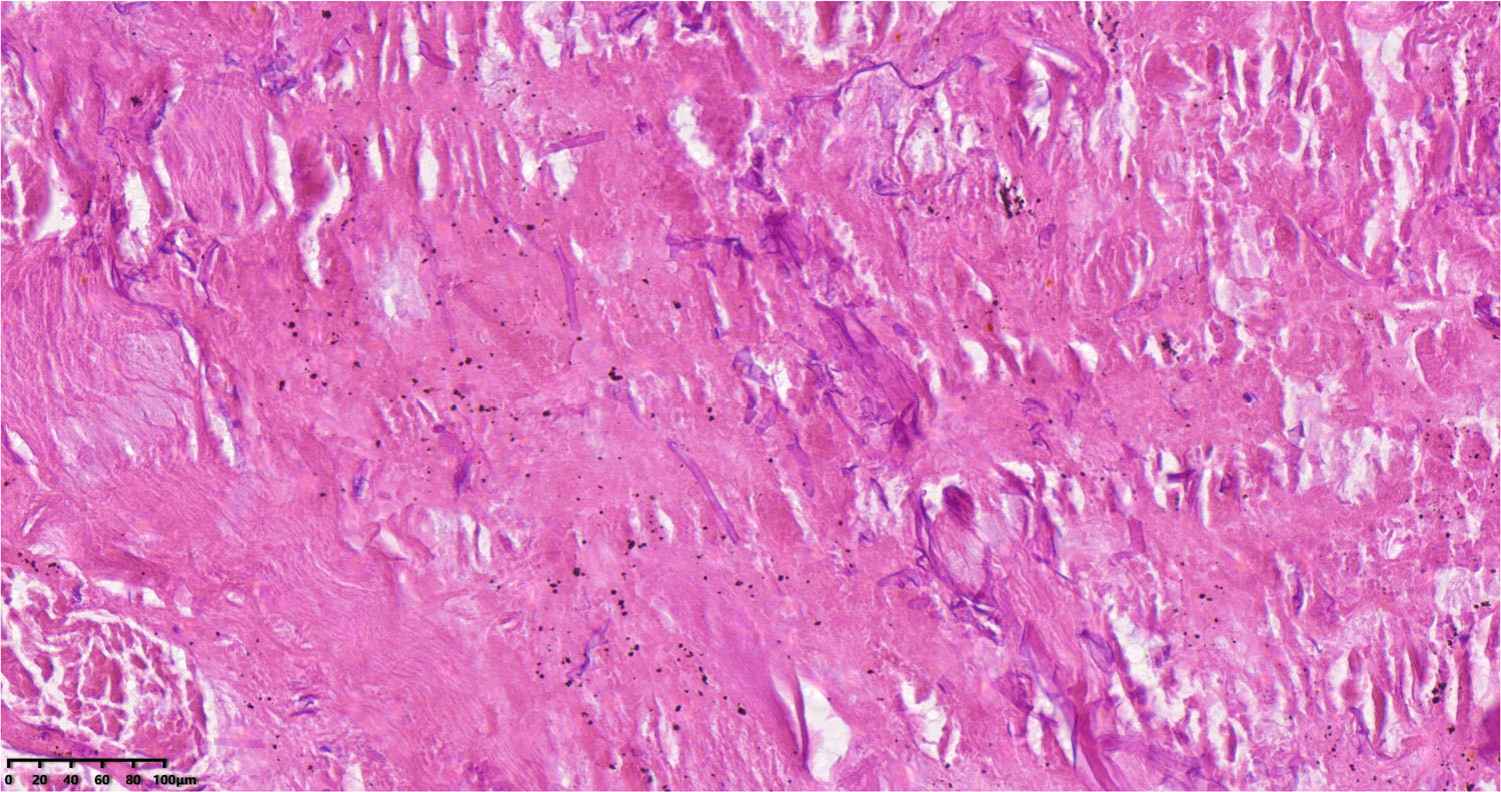
Necrotic tissue shows hyphae (HE stain X40).

**Figure 3:**
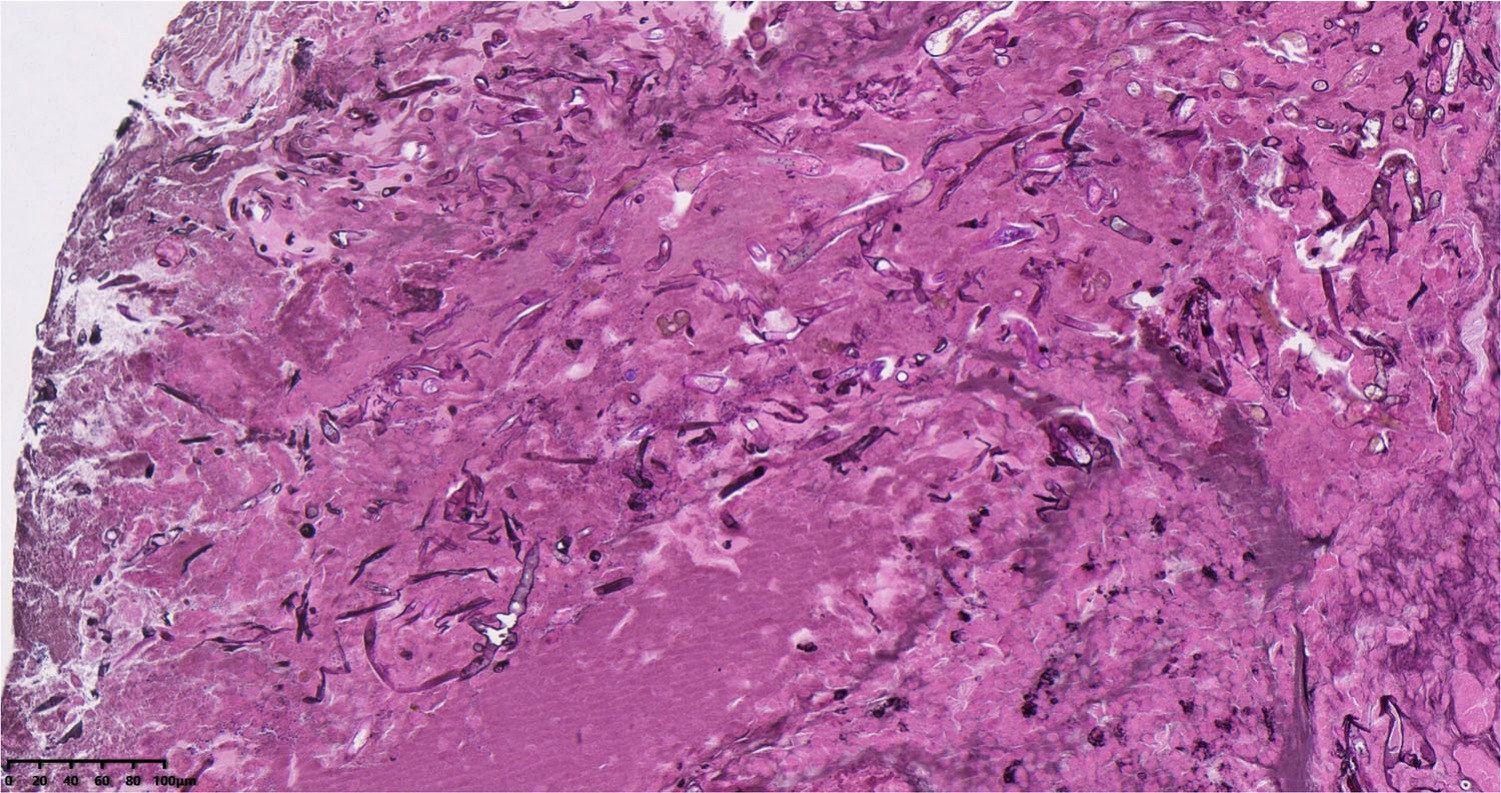
Hyphae and spores observed in the necrotic tissue (silver stain X40).

After being admitted to the hospital on November 1, 2022, the patient received symptomatic and supportive care, including blood glucose control, anti‐infection treatment with piperacillin and tazobactam sodium 4.5 g Q8h, fluid supplementation, acid inhibition, inhibition of pancreatic enzymes, and blood pressure management. The patient was administered meropenem (2 g, Q8h) combined with vancomycin (1000 mi = Q12h) for anti‐infection treatment on November 5, 2022. Furthermore, repeated culture and drug sensitivity tests were conducted to determine the etiology. The patient was in a deep coma and did not respond to vocal stimuli on November 6, 2022. The central venous pressure was measured at 3 cmH_2_O, and the blood oxygen saturation fluctuated in 80%. Endotracheal intubation and ventilator‐assisted ventilation were performed after consultation with the anesthesiology department. The patient continued to receive blood glucose regulation, anti‐infection treatment with meropenem (2 g, Q8h) combined with vancomycin (1000 mg, Q12h), fluid supplementation, acid inhibition, and inhibition of pancreatic enzymes. Considering the high‐density shadow of the patient's left retrobulbar, and after multidepartment consultation, the infection focus of adjacent brain tissue was obviously expanded. Following communication with the patient's family and the signing of informed consent for the surgery, the left eyeball was enucleated in conjunction with peribulbar debridement and left sinus debridement under general anesthesia. There was a localized adhesion above the left eyeball during the surgery. When the eyeball was enucleated, it was found that the nasal bulbar wall and orbital medial wall of the left eye were black, the left nasal cavity and nasal mucosa were black necrotic, and no pus was drawn from the nasal sinus. After the surgery, the possibility of a fungal infection was discussed and analyzed, and cerebrospinal fluid pathogenic metagenomics (NGS) detection was performed. NGS test report on November 8, 2022: *Rhizopus oryzae* infection, no positive cocci or virus infection, no drug resistance gene; on November 9, 2022, isavuconazonium sulfate for injection (200 mg, Q8h) was used. The pathology report on November 14, 2022, stated that fungal hyphae and spores were found in the submitted tissues, indicating a fungal infection (Figures 1, 2, 3).

### 2.3 Outcome

At 23:00 on November 10, 2022, the heart rate suddenly dropped to 60 beats/min, and the blood pressure dropped to 46/29 mmHg. Adjuvant treatment included cardiopulmonary resuscitation, intravenous atropine, and epinephrine. The patient received electric defibrillation after developing ventricular fibrillation, but the rescue was ineffective, and he was pronounced clinically deceased at 23:48.

## 3 DISCUSSION

Mucor is common, but mucormycosis is rare. Mucormycosis is a rare but potentially serious disease caused by naturally occurring “Mucor” fungi in the environment. When the body's immune system is weakened, Mucor enters the upper respiratory tract, bronchi, alveoli and skin through the nasal cavity, oral cavity or damaged skin. Due to the immune deficiency, phagocytic cells are unable to engulf the pathogen, allowing Mucor to colonise the body and cause inflammation.^5^ In China, Lin et al. conducted a retrospective study on 390 cases of mucormycosis.^5^ The majority of patients were male (61.3%), and diabetes was the most common susceptibility factor (37.2%). Pulmonary mucormycosis (42.1%) was the most prevalent disease, followed by skin infections (21.0%). Worldwide, the incidence rate of *Mucor* infection in diabetic patients is between 17% and 88%.^6^

ROCM is more prevalent in patients with diabetic ketosis or acidosis.^1^ *Mucor* has a particular affinity for and invasiveness for blood vessels, which can invade the blood vessels at the infected sites. When fungal hyphae invade blood vessels, a fibrin reaction is triggered, leading to the formation of a thrombus or aneurysm, thus resulting in tissue ischemia and infarction. Infarction will produce black necrotic scars in the nasal cavity, oral cavity, and visage, which are the features and manifestations of nasal‐cerebral mucormycosis. Vascular occlusion also hinders the penetration of antifungal medications, which favor the growth of mucor, creating a vicious cycle.^7^ According to previous studies, the susceptibility of diabetic patients to mucormycosis depends on the increase of free iron in the host's serum. Parminder et al. demonstrated that fungi had a low dependence on free iron.^1^

The initial clinical symptoms of ROCM are atypical and include a runny nose, nasal congestion, headache, fever, facial edema, and low morale. As the disease rapidly progresses, it can manifest as an intraorbital and intracranial invasion. Typical clinical symptoms include combined orbital inflammation, eyelid edema or ptosis, unilateral periorbital facial pain, headache, acute eye movement function changes, and acute visual loss,^8^ which are consistent with the clinical manifestations of this case, even spreading to the brain and causing intracranial invasive infection, embolic necrosis, and finally hemiplegia and loss of consciousness.^1^ According to the Global Guidelines for the Diagnosis and Treatment of Mucormycosis published in 2019 by Lancet Infection Disease, the total mortality rate of mucormycosis ranges from 40% to 80%,^9^ depending on the underlying condition of the host, the infecting species, and the site of infection. The mortality rate of patients infected with *Cunninghamella* (77%) is often higher than that of those infected with *Lichtheimia* (35%), *Rhizopus* (39%), *Mucor* (41%), and *Rhizomucor* (47%). Additionally, patients with hematological malignancies, HSCT (hematopoietic stem cell transplantation), and severe burns have an extremely unfavorable prognosis. If there is dissemination of the infection, particularly involving the craniocerebral, the mortality rate is as high as 80%.^10^

The patient was admitted to the hospital due to a diabetic hyperosmotic coma with acute pancreatitis as the underlying disease. The patient had proptosis of the eyes, orbital inflammation, ptosis of the left upper eyelid, and a fixed eyeball that was unable to rotate, which were retrospectively identified as typical manifestations of ROCM. He had a fever at the initial stage, antibiotics have a poor anti‐infection effect, and ultimately he developed an intracranial infection and loss of consciousness. Therefore, when encountering susceptible people with local eschar necrosis or other typical manifestations, it is highly recommended to consider *Mucor* infection highly suspected. In terms of imaging, early CT examination did not show any features or manifestations, and there was no invasion of the sinus. Four days after admission, an orbital CT examination was performed again to show that the entire group had paranasal sinusitis that had invaded the left turbinate bone. Five days after the admission, a sinus CT showed that the entire group had sinusitis and a maxillofacial infection. Craniocerebral MRI revealed involvement manifestations of the craniocerebral, such as bilateral frontal lobe cerebral infarction, and arterial occlusion, indicating a rapid development of the disease. Computed tomography, or magnetic resonance imaging, is a commonly used diagnostic method. ROCM can also be diagnosed by molecular detection, species identification, polymerase chain reaction^11^ and other methods in addition to the gold criteria of diagnosis‐histopathological examination and fungal culture.^12^

The incidence of ROCM is increasing worldwide. Under the influence of novel coronaviruses, the large‐scale infection cases reported in India have drawn widespread attention. However, there is presently a treatment with high efficacy and low toxicity. Early diagnosis, antifungal treatment, and surgical debridement can improve the prognosis.^1^ However, the surgery must be conducted based on the patient's tolerance. If the patient has eye or brain involvement or a compromised immune system, the necessity and timing of surgery remain ambiguous and require further discussion. Antifungal drugs mainly include amphotericin B and its liposomes, posaconazole, isaconazole, and so forth.^13^ The ESCMID/ECMM guidelines^13^ recommend liposomal amphotericin B (B Ⅱ) and isaconazole (B Ⅱ) for first‐line treatment and posaconazole (C Ⅲ) for maintenance treatment. Due to the broad antibacterial spectrum, fewer drug interactions, stable blood concentration, low incidence of adverse reactions, and excellent long‐term safety of isavuconazole, it is also used as a preventive medication for high‐risk populations. However, further clinical research is required to verify its preventive efficacy.^14^ In this case, the patient was admitted, ketone levels were actively lowered to control blood glucose, pancreatic enzymes were stabilized to treat the underlying disease, and local surgical debridement was performed. Despite treatment with isavuconazole as an antifungal, the infection had already spread to the central nervous system, and the time and dosage of administration may be one of the factors contributing to the poor prognosis.

Current research indicates that factors associated with poor prognosis include: delay in antifungal treatment, craniocerebral involvement, and visual impairment in the early stage.^1^ The poor prognosis of this case was attributed to: ① Early diagnosis is not appropriate. In reviewing the medical history of the patient, the initial symptoms were fatigue and a poor appetite. However, the first hospital only conducted head magnetic resonance imaging to rule out craniocerebral diseases and discharged the patient without examining his blood glucose, resulting in a lost opportunity to control glucose and correct acid. Although the patient had no history of diabetes, his symptoms were consistent with hyperosmolar coma, and his blood glucose levels and islet function should be detected as soon as possible. ② Antifungal medication was used relatively late. After admission on November 1, bacteria were repeatedly detected in cerebrospinal fluid culture, blood culture, and urine culture. The effect of strong antibiotics was unsatisfactory, and the patient's symptoms continued to worsen, so other infection factors should be considered in due time. Until the 9th day after admission, NGS testing revealed an infection with mucorales, and treatment with isavuconazole 200 mg Q8h was started. Despite this, the antifungal treatment window was relatively late and failed to effectively curb the growth of mucor. ③ Rapidly developing lesions. Mucormycosis is the most prevalent cause of orbital apex tissue involvement among all fungal infections.^6^ The patient was admitted with ocular proptosis, which may have been caused by a retrobulbar inflammatory reaction or involvement of the orbital apex tissue. The involvement of the orbital apex tissue is often attributed to the spread of infection from the sphenoid sinus, which also formed an important intracranial pathway. At that time, the possibility of ischemia and necrosis of local mucosal blood vessels and surrounding tissues was not ruled out. As the disease progressed, *Mucor* first infiltrated the orbit, then gradually spread along the blood vessels to the face and nose, and eventually entered the brain, resulting in bilateral frontal lobe cerebral infarction and multiple arteriovenous involvement. Once the lesion affects the craniocerebral area, the survival rate of patients is extremely low^15^; ④ Poor control of underlying diseases. After admission, the patient's blood glucose fluctuated greatly, resulting in ketoacidosis, electrolyte disturbance, poor control of pancreatic enzymes, gradual immune dysfunction, and multiple organ damage, which created a favorable environment for the growth of mucor. In addition, preoperative craniocerebral MRI revealed bilateral frontal lobe cerebral infarction, arterial occlusion, and other manifestations of craniocerebral involvement. The feasibility of surgical treatment is also a factor that must be considered.

## 4 CONCLUSION

These results indicate that mucormycosis is relatively rare, challenging to diagnose and treat, and has a poor prognosis with a high mortality rate. Familiarity with the characteristics of mucormycosis, early diagnosis combined with typical manifestations, timely and effective surgery, and antifungal treatment are crucial for improving the survival rate and prognosis.

## AUTHOR CONTRIBUTIONS

Ying Xie designed research, collected data, and finally revised the manuscript. Jie‐Qiong Ding analyzed the data and wrote a manuscript. All authors have read and approved the final manuscript.

## CONFLICT OF INTEREST STATEMENT

The authors declare no conflict of interest.

## ETHICS STATEMENT

This study was conducted in accordance with the declaration of Helsinki. This study was conducted with approval from the Ethics Committee of Shanxi Provincial People's Hospital. A written informed consent was obtained from legal guardians of all participants. Consent for publication was obtained from every individual whose data are included in this manuscript.

## Data Availability

The data sets used during the current study available from the corresponding author on reasonable request.
